# Correction: Reference rate for post-tonsillectomy haemorrhage in Australia—A 2000–2020 national hospital morbidity database analysis

**DOI:** 10.1371/journal.pone.0321297

**Published:** 2025-03-27

**Authors:** The PLOS One Staff

The caption for Fig 5 has been incorrectly included as main text. Please see the complete, correct Fig 5 caption here.

**Fig 5:**
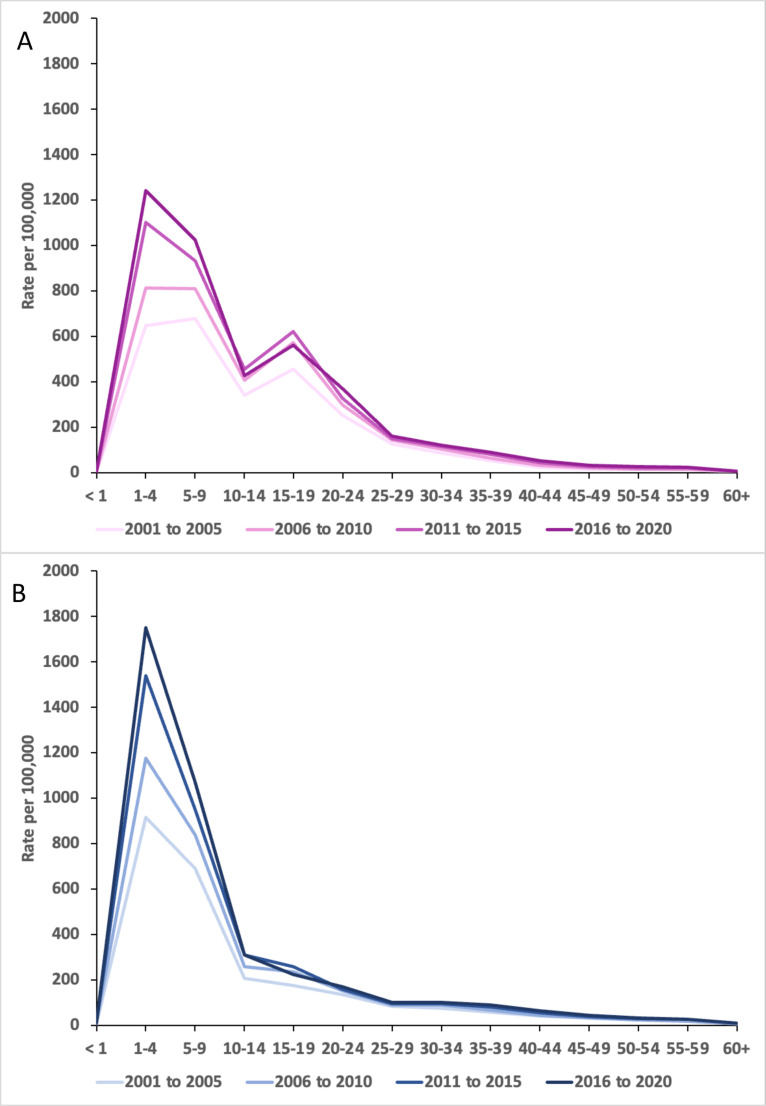
Age-specific incidence of tonsillectomy procedures for males and females, 2000–01 to 2019–20. Age-specific incidence rate averaged over 5-year periods from 2000-01 to 2019-20 for females (A) and males (B). Rate expressed in procedures per 100,000 person-years. Data obtained from the National Hospital Morbidity Database.

The ORCID iD is missing for the second author. Please see the author’s ORCID iD here:

- Author Martin Forer’s ORCID iD is 0000-0001-6283-3970 (https://orcid.org/0000-0001-6283-3970).

The publisher apologizes for the errors.
