# Supplementary material for: Reference rate for post-tonsillectomy haemorrhage in Australia—A 2000–2020 national hospital morbidity database analysis
Source: PLoS One. 2022 Aug 25;17(8):e0273320. doi: 10.1371/journal.pone.0273320 (PMC9409536; doi:10.1371/journal.pone.0273320)
Supplement: S1 Table — Data obtained from the National Hospital Morbidity Database for the period 1 July 2000 to 30 June 2020. PTH = post-tonsillectomy haemorrhage; CI = confidence interval; SD = standard deviation. Annual data are presented as absolute number of episodes of PTH and tonsillectomy procedures. PTH defined by re-operations for haemostasis. Annual PTH rates are calculated by dividing the number of PTH with the number of tonsillectomy procedures. The annual mean and standard deviation were calculated for the entire study period. (DOCX) [file pone.0273320.s001.docx]

**S1 Table. Annual rates of post-tonsillectomy haemorrhage in Australia, 2000-01 to 2019-20.**

| **Year** | **PTH** | **Tonsillectomy procedures** | **PTH rate (%)** | **95% CI** |
| --- | --- | --- | --- | --- |
| **2000-01** | 468 | 30,165 | 1.6 | (1.41, 1.70) |
| **2001-02** | 514 | 33,833 | 1.5 | (1.39, 1.66) |
| **2002-03** | 481 | 33,060 | 1.5 | (1.33, 1.59) |
| **2003-04** | 494 | 32,581 | 1.5 | (1.39, 1.66) |
| **2004-05** | 548 | 33,638 | 1.6 | (1.50, 1.77) |
| **2005-06** | 566 | 35,303 | 1.6 | (1.47, 1.74) |
| **2006-07** | 643 | 36,661 | 1.8 | (1.62, 1.89) |
| **2007-08** | 707 | 42,114 | 1.7 | (1.56, 1.81) |
| **2008-09** | 759 | 47,483 | 1.6 | (1.49, 1.72) |
| **2009-10** | 754 | 47,645 | 1.6 | (1.47, 1.70) |
| **2010-11** | 816 | 50,128 | 1.6 | (1.52, 1.74) |
| **2011-12** | 821 | 51,098 | 1.6 | (1.50, 1.72) |
| **2012-13** | 933 | 53,278 | 1.8 | (1.64, 187) |
| **2013-14** | 1,023 | 54,662 | 1.9 | (1.76, 1.99) |
| **2014-15** | 1,053 | 56,506 | 1.9 | (1.75, 1.98) |
| **2015-16** | 1,044 | 62,801 | 1.7 | (1.56, 1.77) |
| **2016-17** | 1,032 | 65,052 | 1.6 | (1.49, 1.69) |
| **2017-18** | 1,090 | 63,521 | 1.7 | (1.62, 1.82) |
| **2018-19** | 930 | 60,661 | 1.5 | (1.44, 1.63) |
| **2019-20** | 715 | 51,367 | 1.4 | (1.29, 1.50) |
| **Mean** | 770 | 47,078 | 1.6 | - |
| **SD** | 215 | 11,641 | 0.1 | - |

Data obtained from the National Hospital Morbidity Database for the period 1 July 2000 to 30 June 2020. PTH = post-tonsillectomy haemorrhage; CI = confidence interval; SD = standard deviation. Annual data are presented as absolute number of episodes of PTH and tonsillectomy procedures. PTH defined by re-operations for haemostasis. Annual PTH rates are calculated by dividing the number of PTH with the number of tonsillectomy procedures. The annual mean and standard deviation were calculated for the entire study period.
