# Supplementary material for: Reference rate for post-tonsillectomy haemorrhage in Australia—A 2000–2020 national hospital morbidity database analysis
Source: PLoS One. 2022 Aug 25;17(8):e0273320. doi: 10.1371/journal.pone.0273320 (PMC9409536; doi:10.1371/journal.pone.0273320)
Supplement: S2 Table — Data obtained from the National Hospital Morbidity Database for the period 1 July 2000 to 30 June 2020. PTH = post-tonsillectomy haemorrhage; CI = confidence interval. *P < 0.0001. (DOCX) [file pone.0273320.s002.docx]

**S2 Table. Comparison of age-specific post-tonsillectomy haemorrhage rates between age groups for each gender.**

|  | **Age** | **PTH** | **Tonsillectomy procedures** | **PTH rate (%)** | **Odds ratio** | **95% CI** | ***P* value** |
| --- | --- | --- | --- | --- | --- | --- | --- |
| **Male** | 1 - 4 | 1,284 | 161,783 | 0.8 | Ref | | |
|  | 5 - 9 | 1,352 | 131,971 | 1.0 | 1.3 | (1.2, 1.4) | * |
|  | 10 - 14 | 676 | 39,578 | 1.7 | 2.2 | (2.0, 2.4) | * |
|  | 15 - 19 | 1,325 | 33,045 | 4.0 | 5.2 | (4.8, 5.6) | * |
|  | 20 - 24 | 1,362 | 24,836 | 5.5 | 7.3 | (6.7, 7.8) | * |
|  | 25 - 29 | 741 | 15,193 | 4.9 | 6.4 | (5.8, 7.0) | * |
|  | 30 - 34 | 625 | 14,573 | 4.3 | 5.6 | (5.1, 6.2) | * |
|  | 35 - 39 | 385 | 11,753 | 3.3 | 4.2 | (3.8, 4.8) | * |
|  | 40 - 44 | 285 | 8,000 | 3.6 | 4.6 | (4.1, 5.3) | * |
|  | 45 - 49 | 159 | 5,554 | 2.9 | 3.7 | (3.1, 4.4) | * |
|  | 50 - 54 | 103 | 4,051 | 2.5 | 3.3 | (2.7, 4.0) | * |
|  | 55 - 59 | 62 | 2,990 | 2.1 | 2.6 | (2.0, 3.4) | * |
|  | 60 + | 107 | 4,609 | 2.3 | 3.0 | (2.4, 3.6) | * |
| **Female** | 1 - 4 | 766 | 108,477 | 0.7 | Ref | | |
|  | 5 - 9 | 1276 | 121,576 | 1.0 | 1.5 | (1.4, 1.6) | * |
|  | 10 - 14 | 820 | 56,167 | 1.5 | 2.1 | (1.9, 2.3) | * |
|  | 15 - 19 | 1637 | 77,661 | 2.1 | 3.0 | (2.8, 3.3) | * |
|  | 20 - 24 | 1034 | 48,184 | 2.1 | 3.1 | (2.8, 3.4) | * |
|  | 25 - 29 | 502 | 23,567 | 2.1 | 3.1 | (2.7, 3.4) | * |
|  | 30 - 34 | 351 | 17,581 | 2.0 | 2.9 | (2.5, 3.3) | * |
|  | 35 - 39 | 222 | 11,355 | 2.0 | 2.8 | (2.4, 3.3) | * |
|  | 40 - 44 | 113 | 6,166 | 1.8 | 2.6 | (2.2, 3.2) | * |
|  | 45 - 49 | 65 | 3,688 | 1.8 | 2.5 | (2.0, 3.3) | * |
|  | 50 - 54 | 53 | 3,019 | 1.8 | 2.5 | (1.9, 3.3) | * |
|  | 55 - 59 | 30 | 2,425 | 1.2 | 1.8 | (1.2, 2.5) | * |
|  | 60 + | 50 | 3,349 | 1.5 | 2.1 | (1.6, 2.8) | * |

Data obtained from the National Hospital Morbidity Database for the period 1 July 2000 to 30 June 2020. PTH = post-tonsillectomy haemorrhage; CI = confidence interval. **P* < 0.0001.
