# Supplementary material for: Reference rate for post-tonsillectomy haemorrhage in Australia—A 2000–2020 national hospital morbidity database analysis
Source: PLoS One. 2022 Aug 25;17(8):e0273320. doi: 10.1371/journal.pone.0273320 (PMC9409536; doi:10.1371/journal.pone.0273320)
Supplement: S3 Table — Data obtained from the National Hospital Morbidity Database for the period 1 July 2000 to 30 June 2020. Data are presented as n (%). Total number of tonsillectomy procedures was calculated by the sum of tonsillectomy procedure codes. ACHI = The Australian Classification of Health Interventions; UPPP = Uvulopharyngopalatoplasty. (DOCX) [file pone.0273320.s003.docx]

**S3 Table. Characteristics of tonsillectomy procedures in Australia.**

| **Variables** | **Tonsillectomy procedures**  **(n = 941, 557)** | |
| --- | --- | --- |
| **Age (years)** |  |  |
| < 1 | 392 | (0.04%) |
| 1 - 4 | 270,264 | (28.7%) |
| 5 - 9 | 253,550 | (26.9%) |
| 10 - 14 | 95,746 | (10.2%) |
| 15 - 19 | 110,708 | (11.8%) |
| 20 - 24 | 73,020 | (7.8%) |
| 25 - 29 | 38,762 | (4.1%) |
| 30 - 34 | 32,155 | (3.4%) |
| 35 - 39 | 23,108 | (2.5%) |
| 40 - 44 | 14,167 | (1.5%) |
| 45 - 49 | 9,242 | (1.0%) |
| 50 - 54 | 7,070 | (0.8%) |
| 55 - 59 | 5,415 | (0.6%) |
| 60 + | 7,958 | (0.8%) |
| **Gender** |  |  |
| Male | 458,233 | (48.7%) |
| Female | 483,310 | (51.3%) |
| **Treatment type** |  |  |
| Day stay | 81,758 | (8.7%) |
| Overnight | 859,797 | (91.3%) |
| **Procedure (defined by ACHI code)** |  |  |
| 41789-01 Tonsillectomy with adenoidectomy | 576,628 | (61.2%) |
| 41789-00 Tonsillectomy without adenoidectomy | 346,419 | (36.8%) |
| 41786-01 UPPP with tonsillectomy | 18,510 | (2.0%) |
| 41797-00 Arrest of haemorrhage following tonsillectomy | - | - |

Data obtained from the National Hospital Morbidity Database for the period 1 July 2000 to 30 June 2020. Data are presented as n (%). Total number of tonsillectomy procedures was calculated by the sum of tonsillectomy procedure codes. ACHI = The Australian Classification of Health Interventions; UPPP = Uvulopharyngopalatoplasty.
