# Supplementary material for: Reference rate for post-tonsillectomy haemorrhage in Australia—A 2000–2020 national hospital morbidity database analysis
Source: PLoS One. 2022 Aug 25;17(8):e0273320. doi: 10.1371/journal.pone.0273320 (PMC9409536; doi:10.1371/journal.pone.0273320)
Supplement: S4 Table — Population data are the mid-year estimated resident population sourced from the Australian Bureau of Statistics (ABS). Absolute tonsillectomy procedure counts are obtained from the National Hospital Morbidity Database for the period 1 July 2000 to 30 June 2020. 95% confidence intervals for incidence rates are estimated using the Poisson regression model. (DOCX) [file pone.0273320.s004.docx]

**S4 Table. Annual incidence of tonsillectomy procedures in Australia, 2000-01 to 2019-20.**

|  |  | **Overall incidence** | | |
| --- | --- | --- | --- | --- |
| **Year** | **Population** | **Tonsillectomy procedures (n)** | **Rate (per 100,000)** | **95% CI** |
| **2000 - 01** | 19,274,701 | 30,165 | 156.5 | (154.7, 158.3) |
| **2001 - 02** | 19,495,210 | 33,833 | 173.5 | (171.7, 175.4) |
| **2002 - 03** | 19,720,737 | 33,060 | 167.6 | (165.8, 169.5) |
| **2003 - 04** | 19,932,722 | 32,581 | 163.5 | (161.7, 165.2) |
| **2004 - 05** | 20,176,844 | 33,638 | 166.7 | (164.9, 168.5) |
| **2005 - 06** | 20,450,966 | 35,303 | 172.6 | (170.8, 174.4) |
| **2006 - 07** | 20,827,622 | 36,661 | 176.0 | (174.2, 177.8) |
| **2007 - 08** | 21,249,199 | 42,114 | 198.2 | (196.3, 200.1) |
| **2008 - 09** | 21,691,653 | 47,483 | 218.9 | (216.9, 220.9) |
| **2009 - 10** | 22,031,750 | 47,645 | 216.3 | (214.3, 218.2) |
| **2010 - 11** | 22,340,024 | 50,128 | 224.4 | (222.4, 226.4) |
| **2011 - 12** | 22,733,465 | 51,098 | 224.8 | (222.8, 226.7) |
| **2012 - 13** | 23,128,129 | 53,278 | 230.4 | (228.4, 232.3) |
| **2013 - 14** | 23,475,686 | 54,662 | 232.8 | (230.9, 234.8) |
| **2014 - 15** | 23,815,995 | 56,506 | 237.3 | (235.3, 239.2) |
| **2015 - 16** | 24,190,907 | 62,801 | 259.6 | (257.6, 261.6) |
| **2016 - 17** | 24,601,860 | 65,052 | 264.4 | (262.4, 266.5) |
| **2017 - 18** | 24,982,688 | 63,521 | 254.3 | (252.3, 256.2) |
| **2018 - 19** | 25,365,745 | 60,661 | 239.1 | (237.2, 241.1) |
| **2019 - 20** | 25,693,267 | 51,367 | 199.9 | (198.2, 201.7) |

Population data are the mid-year estimated resident population sourced from the Australian Bureau of Statistics (ABS). Absolute tonsillectomy procedure counts are obtained from the National Hospital Morbidity Database for the period 1 July 2000 to 30 June 2020. 95% confidence intervals for incidence rates are estimated using the Poisson regression model.
