# Supplementary material for: Reference rate for post-tonsillectomy haemorrhage in Australia—A 2000–2020 national hospital morbidity database analysis
Source: PLoS One. 2022 Aug 25;17(8):e0273320. doi: 10.1371/journal.pone.0273320 (PMC9409536; doi:10.1371/journal.pone.0273320)
Supplement: Attachment — Submitted filename: Response to reviewers.docx [file pone.0273320.s005.docx]

**Reference rate for post-tonsillectomy haemorrhage in Australia - A 2000-2020 national hospital morbidity database analysis**

Dear Dr Sethu Thakachy Subha,

Thank you for reviewing our manuscript and for providing us with Dr Haytham Kubba’s comments.

Please find our responses in the table below. The line numbers that are mentioned in our responses refer to the mark-up copy of the manuscript.

Kind regards,

Jonathan Li

|  | **Reviewer comments** | **Author responses** |
| --- | --- | --- |
| 1. | I enjoyed reading this paper. Tonsillectomy is so common, and bleeding such a common and serious complication, that it is perhaps surprising that so few national datasets have been published like this (the authors may wish to check out a new paper from the UK which probably wasn't available when they wrote the manuscript: <https://doi.org/10.1111/coa.13707>). | Thank you for your kind comment and for bringing to our attention the recent UK paper by Keltie et al (2021). Their comprehensive national hospital dataset analysis on paediatric tonsillectomy procedures also reports on a national return to theatre rate. Interestingly, their reported rate for the UK (1.2%) is comparable to the rate we have calculated for Australia (1.0%).  We have amended the table (now Table 3) “Summary of population studies” in our discussion section to include the result of this study. |
| 2. | This paper is a useful addition to the literature and I would be keen to see it published. Is there any comment the authors could make on data completeness in the NHMD (ie the proportion of hospital episodes for which a complete and valid data return is made for the NHMD)? | Thanks for raising the question on data completeness which is an important point to mention. Data completeness of the NHMD datasets can be affected by the accuracy of the administration and reporting processes of the individual hospitals as well as the quality of the processing of data at the State and Territory level.  According to AIHW’s data Quality Statement, one of its functions is to work with each State and Territory to set and maintain national data standards and to continually improve the quality of health administrative data. It also states that the AIHW undertakes “extensive validations on the receipt of data” and the data is cross checked with other data sets where possible  (<https://meteor.aihw.gov.au/content/724188>).  We have now revised the ‘Strengths and limitations’ paragraph to include the issue of data completeness (line 555 to 560). |
|  | Also, could the authors comment on the possibility of miscoding (eg post-tonsillectomy haemorrhage being coded under some other "arrest of haemorrhage" code)? | Thank you for raising this discussion point.  There are three other “arrest of haemorrhage” ACHI procedure codes listed in the ENT category. These codes are 41656-00, 41677-00 and 41680-00 and are specific for the condition of epistaxis.  Whilst human error in coding can occur, we anticipate miscoding to be rare occurrences. |
| 3. | I find Table 1 misleading and unhelpful. I don't think anyone is interested in what proportion of all the bleeds occur in a particular age group because of course the number of tonsillectomies done varies so much between age groups that this proportion is meaningless. Table 1 would be more useful if it presented the haemorrhage rate for each age group. The same is true in the text: "the age group that had the highest proportion of PTH being the 15 to 19 years age group (19.2%) followed by the 5 to 9 years age group (17.1%)". More bleeds occur at these ages because more tonsillectomies are done at these ages. The reader is easily misled by this into thinking these are the ages with the highest bleed rates which is not the case. The actual bleed rates seem to rise smoothly through adolescence and early adulthood, so the highest rates are between roughly 15 and 35 years of age. | Thank you for the constructive comment.  Table 1 contains the absolute number and percentage total of the PTH episodes recorded for each age group. We agree that the table may be misinterpreted as the age-specific PTH rates especially given this is the main narrative of the paper. We have therefore removed this table and its accompanying text (Lines 154 to 157).  The original Table 1 has been revised to include only the ‘Tonsillectomy procedures’ column and is now placed under Supporting information as S3 Table. S3 Table is cited in the result section ‘Epidemiology of tonsillectomy in Australia’ (Line 273). |
| 4. | I am not sure Table 2 adds much either. I would be happy just to see the statement in the text that there is no discernible time trend in the overall numbers and then move on. Figure 1a gives us the information anyway. Table 3 is much more interesting, with the adult and paediatric data presented separately, together with Figure 1b. | Thank you for your suggestion. We have removed Table 2 from the text and simplified the wording of the results section ‘Time-trends of post-tonsillectomy rates’. However, we have kept the table in the Supporting information (S1 Table) as we believe the absolute values used to calculate the PTH rates in Figure 1A may be of interest to some readers. We have also added two rows to include values for Mean and SD.  S1 Table is now mentioned in the results (line 159) and in the caption for Figure 1A (line 189). |
| 5. | Figure 2 and Table 4 are very interesting, with the difference in bleed rates between males and females being quite marked. In fact the biggest difference occurs in the young adult years which happens to be when the highest overall bleed rates occur, and the male bleeds largely account for the increase in the overall bleed rates at this age. Personally, I would combine this information with a table of overall bleed rates in table 1 to tell this story more clearly. I don't think Table 5 adds much: do we really need this in addition to tables 4 and 6? | We agree Table 5 does not add much to the flow of the main text. However, we feel this table is useful for further emphasising the difference in the odds of PTH between age groups for each gender. Accordingly, we have moved Table 5 from the main text to the Supporting information section (S2 Table). The accompanying paragraph (line 228 to 236) has been reworded to reflect this change. |
| 6. | Discussion and references are comprehensive. So my only real gripes are about the sheer volume of information presented in tables which is a bit overwhelming and serves to obscure the important messages in the paper: trim these a little and the more streamlined paper will be easier to follow. | Your feedback and suggestions to improve the paper has been greatly appreciated. |
|  |  | Other revisions:   - Conclusion has been revised to improve readability. - The new Table 1 and S1 Table now include rows for Mean and SD. - The numbering of the tables has been updated. There are now 4 tables in the main text and 4 tables in the Supporting information section. - The Captions for the Tables in the Supporting information has been updated. - Reference numbering and list has been updated:   - Hyperlink of reference 17 corrected. (line 667)   - paper by Keltie et al (2021) is moved from No. 52 to No. 24.   - Reference 53 replaced. - Figures are unchanged except for minor edit to colour scheme of Figure 2 to match colour scheme of Figure 5. - Other minor rewording and word changes:   - Line 105   - Line 172   - Line 202   - Line 204   - Line 207 (table title)   - Line 220   - Line 298   - Line 304   - Line 325 |
